# Fast “Feast/Famine” Cycles for Studying Microbial Physiology Under Dynamic Conditions: A Case Study with *Saccharomyces cerevisiae*

**DOI:** 10.3390/metabo4020347

**Published:** 2014-05-15

**Authors:** Camilo A. Suarez-Mendez, Andre Sousa, Joseph J. Heijnen, Aljoscha Wahl

**Affiliations:** 1 Department of Biotechnology, Delft University of Technology, Julianalaan 67, 2628 BC Delft, The Netherlands; E-Mail: j.j.heijnen@tudelft.nl; 2 Kluyver Center for Genomics of Industrial Fermentation, P.O. Box 5057, 2600 GA Delft, The Netherlands

**Keywords:** feast/famine perturbation, yeast cultivation, dynamic metabolic response, ATP paradox, *in vivo* kinetics

## Abstract

Microorganisms are constantly exposed to rapidly changing conditions, under natural as well as industrial production scale environments, especially due to large-scale substrate mixing limitations. In this work, we present an experimental approach based on a dynamic feast/famine regime (400 s) that leads to repetitive cycles with moderate changes in substrate availability in an aerobic glucose cultivation of *Saccharomyces cerevisiae*. After a few cycles, the feast/famine produced a stable and repetitive pattern with a reproducible metabolic response in time, thus providing a robust platform for studying the microorganism’s physiology under dynamic conditions. We found that the biomass yield was slightly reduced (−5%) under the feast/famine regime, while the averaged substrate and oxygen consumption as well as the carbon dioxide production rates were comparable. The dynamic response of the intracellular metabolites showed specific differences in comparison to other dynamic experiments (especially stimulus-response experiments, SRE). Remarkably, the frequently reported ATP paradox observed in single pulse experiments was not present during the repetitive perturbations applied here. We found that intracellular dynamic accumulations led to an uncoupling of the substrate uptake rate (up to 9-fold change at 20 s.) Moreover, the dynamic profiles of the intracellular metabolites obtained with the feast/famine suggest the presence of regulatory mechanisms that resulted in a delayed response. With the feast famine setup many cellular states can be measured at high frequency given the feature of reproducible cycles. The feast/famine regime is thus a versatile platform for systems biology approaches, which can help us to identify and investigate metabolite regulations under realistic conditions (e.g., large-scale bioreactors or natural environments).

## 1. Introduction

In the natural environment, but also at large scale industrial cultivation, organisms are exposed to rapid dynamic conditions [1], which are repetitive [2]. Relevant cultivation parameters, like dissolved oxygen, substrate concentration, pH and temperature do vary in large scale bioreactors [3] because of long mixing times in the order of tens of seconds to minutes [2]. The range of substrate concentrations in large reactors depends on the feed solution concentration (for glucose up to 600 gL^−1^) to very low concentrations (in the order of mg/L) in areas far away from the feeding inflow resulting in fluctuating situations of feast to famine [2]. Thus, microorganisms in the bioreactor experience rapid changing environments that may have detrimental effects such us decrease in product and biomass yield compared to steady-state conditions with the same, but continuous, supply of substrate [1,4]. Substrate fluctuations, depending on the frequency and magnitude, may decrease the biomass yield of *S. cerevisiae* up to 25% [5]. Under such rapid dynamic conditions strong metabolic flux changes are expected as these depend on the extracellular substrate concentration.

In order to reproduce such a dynamic environment a number of approaches have been applied: 1- step and pulse perturbations of glucose limited cultivations [1,5,6,7,8,9], and 2- periodic perturbations [10]. Whereas metabolic stationary approaches aim to quantify intracellular fluxes, non-stationary experiments aim to identify reaction kinetic properties like maximal *in vivo* rates, substrate affinities and allosteric inhibition [11,12]. In this work, we present a complete description of the yeast’s metabolic response to a stimulus-response experiment referred to as feast/famine by which, the microorganisms are exposed to repetitive changes in substrate availability as a result of a block-wise feeding regime in a time window of seconds to minutes. With this setup, we focus on the short-term response by monitoring the *in vivo* metabolic activity (intra and extracellular concentrations and fluxes) during cycles of 400 s. At this short time-scale, it can be assumed that the metabolic fluxes are mainly controlled by metabolite interactions, while enzyme concentrations remain constant over the cycles [13,14].

## 2. Results and Discussion

### 2.1. The Feast/Famine Setup is a Robust Dynamic System for Metabolic Studies

**Figure 1:**
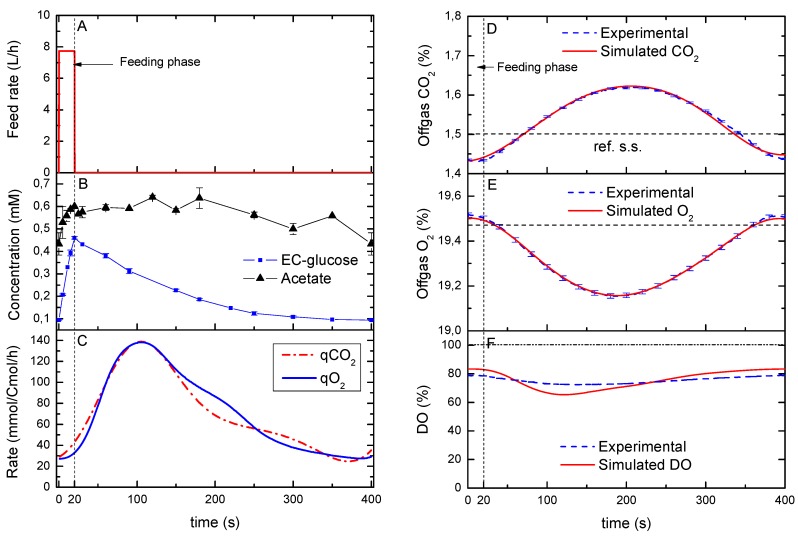
(**A**) Feeding rate during one cycle; (**B**) Measured extracellular concentration of glucose (EC-glucose, squares) and acetate (triangles), error bars represent the standard error of duplicate determination during two subsequent cycles. (**C**) Model-based estimation of the qO_2_ and qCO_2_; (**D**–**F**) Dynamics of the on-line measurements (off-gas CO_2_ and O_2_; DO, dissolved oxygen) during one cycle in comparison to the model simulation (using the rates in C). The horizontal dashed line in D and E represents the reference steady state. The vertical dotted line represents the end of the feeding phase.

**Figure 2:**
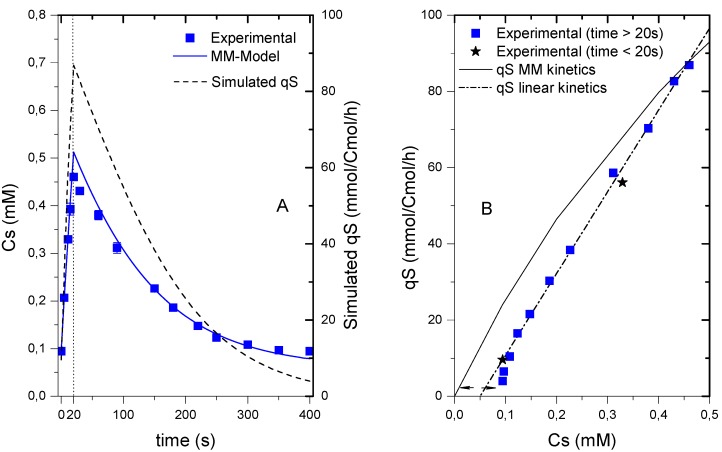
Dynamics of the residual glucose concentration and uptake rate during the cycle. (**A**) Measured extracellular glucose concentration (Cs, closed squares) and Simulated extracellular glucose concentration (solid line) using a MM model (Km = 1mM, q_S,max_ = 279 mmolCmol^−1^h^−1^); Dynamics of the specific uptake rate (q_S_) in time (dashed line); (**B**) Comparison of the experimental q_S_ (from interpolation) as a function of C_S_ (extracellular glucose concentration) to a hyperbolic function (solid line) and a linear function (dot-dashed line). The vertical dotted line represents the end of the feeding phase.

**Figure 3:**
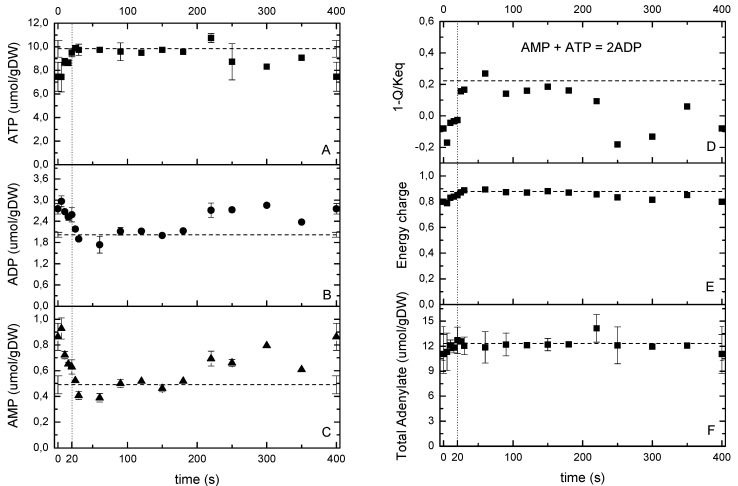
(**A**–**C**) ATP, ADP and AMP measurements during the cycle. (**D**) Thermodynamic driving force for adenylate kinase. (**E**) energy charge and (**F**) total adenylate pool over the cycle (right). The dashed line represents the reference measurement during the chemostat phase (D = 0.1 h^−1^), error bars represent the standard error of duplicate determination during two subsequent cycles. The vertical dotted line represents the end of the feeding phase.

**Figure 4:**
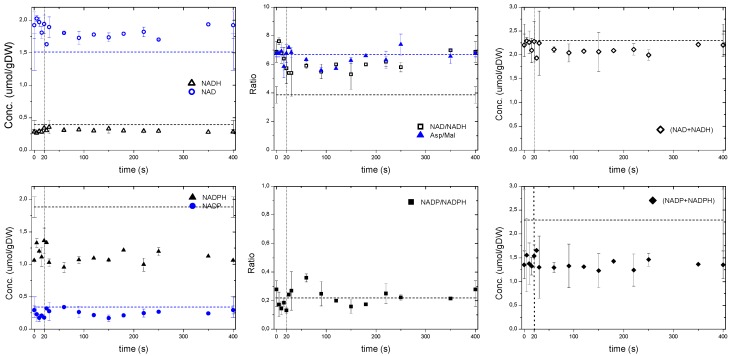
Whole cell measurements of NAD, NADH, NADP, NADPH as well as ratios based on sensor reactions. The dashed line represents the reference measurement during the chemostat phase (D = 0.1 h^−1^), error bars represent the standard error of duplicate determination during two subsequent cycles. The vertical dotted line represents the end of the feeding phase

**Figure 5:**
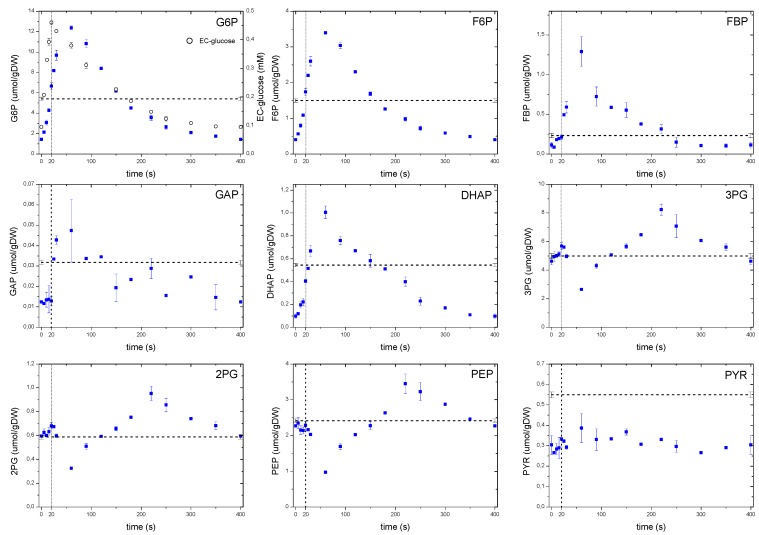
Concentration measurements of glycolytic metabolites during the feast/famine cycle. The dashed line represents the reference steady-state (D = 0.1 h^−1^), error bars represent the standard error of duplicate determination during two subsequent cycles. The vertical dotted line represents the end of the feeding phase.

**Figure 6:**
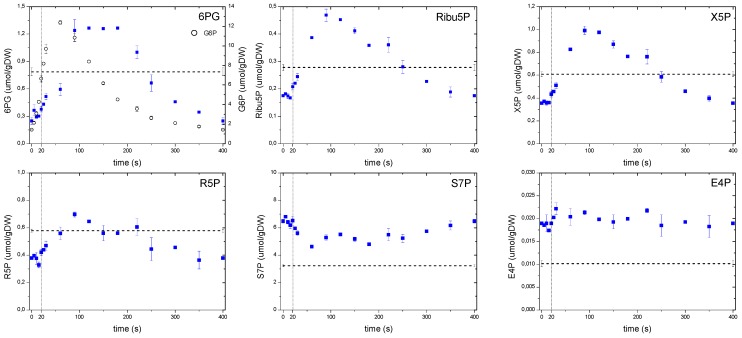
Concentration measurements of the PPP metabolites during the feast/famine cycle. The dashed line represents the reference steady-state (D = 0.1 h^−1^), error bars represent the standard error of duplicate determination during two subsequent cycles. The vertical dotted line represents the end of the feeding phase.

**Figure 7:**
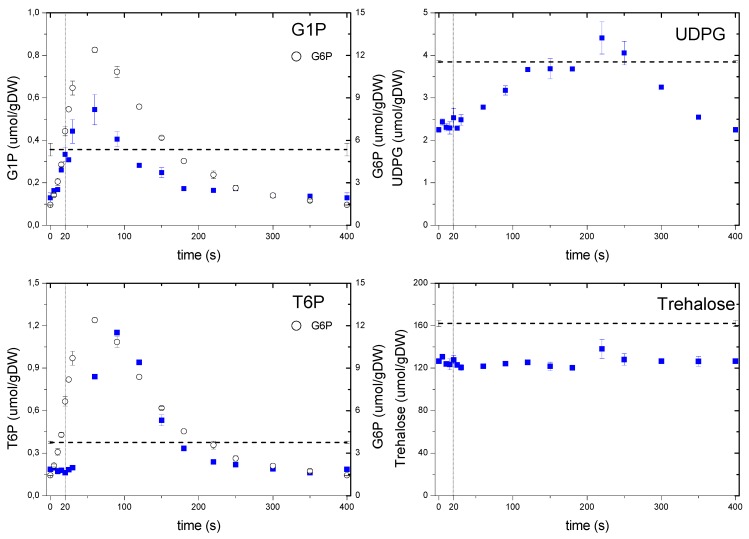
Concentration measurements of storage metabolites during the feast/famine cycle. The dashed line represents the reference steady-state (D = 0.1 h^−1^), error bars represent the standard error of duplicate determination during two subsequent cycles. The vertical dotted line represents the end of the feeding phase.

**Figure 8:**
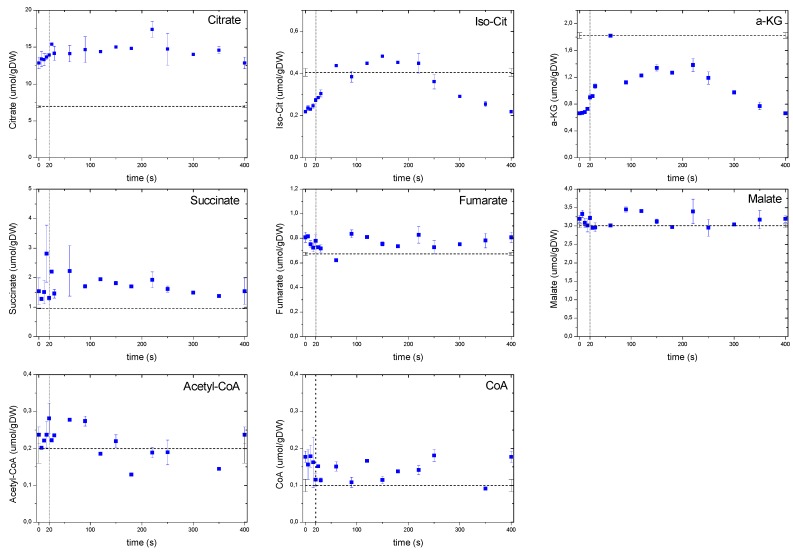
Concentration measurements of the TCA metabolites during the feast/famine cycle. The dashed line represents the reference steady-state (D = 0.1 h^−1^), error bars represent the standard error of duplicate determination during two subsequent cycles. The vertical dotted line represents the end of the feeding phase.

The Feast/famine experimental setup is designed to produce regimes of high and low substrate availability by means of a block-wise addition of the feed. In this type of regime, the feed is only active during a short period of time (feast) where a desired maximal concentration is reached. The feeding phase is followed by a period of no feed (famine) in which the cells take up the substrate. The length of both periods (*i.e.*, the cycle time, in this case 20 s feeding and 380 s of no feed) depend on the desired range of substrate concentration and is dictated by the average growth rate at which the culture is wanted to grow (see Experimental section for further details). We obtained reproducible patterns about 3 to 4 h after starting the block-wise feeding regime (about two fifths of the generation time) as indicated by the off-gas (CO_2_ and O_2_) and DO measurements. In addition, duplicate measurements of intra and extracellular metabolite concentration during two successive cycles (see experimental section) were taken to check the reproducibility of the cycles. The measurements (Figure 1, Figure 2, Figure 3, Figure 4, Figure 5, Figure 6, Figure 7 and Figure 8) suggest a high reproducibility of the cycles and the sampling protocol (deviations were in the order of 5%).

### 2.2. Reconstruction of Specific O_2_ and CO_2_ Rates during a Cycle

The specific carbon dioxide production rate (qCO_2_) and oxygen uptake rate (qO_2_) that were calculated following the approach described by Bloemen, *et al*. [15], showed a similar dynamic profile (Figure 1C) with an average RQ of 1.04. Since the profile of the reconstructed qO_2_ and qCO_2_ did not match the extracellular glucose, it may not be possible to assume a metabolic quasi-steady state between these rates. In other words, whereas the glucose uptake rate is expected to peak with the maximum residual glucose concentration (*i.e.*, maximum uptake flux), the off-gas rates showed clear delays and a less sharp profile (Figure 1D,E). Nevertheless, the reconstruction process resulted in simulated off-gas concentrations (Figure 1D,E) that were in agreement with the online off-gas measurements. On the other hand, the reconstruction of the bioreactor-DO deviated from the one observed experimentally, suggesting that the reconstructed dynamics were more drastic than the measured one (Figure 1F).

This deviation can be explained by drifts due to membrane fouling of the pO_2_ probe during a long cultivation (about 150 h) [16,17], which leads to a changed calibration line and altered probe dynamics. The dissolved oxygen decreased with an increasing glucose supply (0–20 s) due to a higher glucose uptake rate. This decreasing pattern continued until about 100 s, after which, it went back to the starting value of 83% (about 0.264 mmol O_2_/L).

### 2.3. The Feast/Famine vs. the Reference Chemostat Cultivation

In order to compare the dynamic rates with those at steady-state, the O_2_ consumption and CO_2_ production in time were integrated over the cycle (400s) using Matlab ^®^ TRAPZ function. The integration resulted in a reconciled qCO_2_ of 73.2 ± 1.4 mmol Cmol^−1^h^−1^ and qO_2_ of 70.4 ± 1.0 mmol Cmol^−1^ h^−1^ per cycle. Although these were nearly identical to the rates observed for a chemostat cultivation at a dilution rate of 0.1 h^−1^ (74.6 ± 5.4 and 70.0 ± 5.4 mmol Cmol^−1^ h^−1^ respectively), the dynamic qCO_2_ and qO_2_ rates changed up to about 4.5-fold during the cycle (Figure 1C). Van Kleeff *et al.* [5] also observed a 1.54-fold increase in the oxygen uptake rate for another *S. cerevisiae* strain (CBS7336) that was grown at an average dilution rate of 0.05 h^−1^ under similar periodic conditions. Based on that observation, they suggested that the microorganisms used that extra respiratory capacity to adapt quickly to changes in the environment without the formation of by-products. In our study, we can considered that the respiratory capacity was sufficient during all time-points of the cycle since no by-products were observed apart from acetate. In addition, the RQ = 1.04 points to the absence of metabolic overflow (e.g., no production of ethanol and glycerol).

With the exception of the acetate secretion rate, we found only small differences between the reconciled rates for the steady state and the feast/famine (Table 1). The increased production of acetate can partially explain the observed slight decrease in biomass concentration (about 5.0%) as compared to the reference chemostat. Dry weight measurements showed a deviation of about 1.0% from the mean even though the samples were taken during three different cycles at different cycle times. Measurements of the intracellular trehalose concentration during the cycle suggest a lower storage content during the feast/famine regime compared to the steady state conditions. As will be discussed later, this may also contribute to the lower biomass yield due to a possible change in the biomass composition.

**Table 1:** Reconciled rates for both steady-state and feast/famine cultures.

Rate	Steady state ^a^	Feast/famine ^b^
Biomass concentration (gDW/L)	3.64 ± 0.16	3.46 ± 0.17
q_S_ (mmol Cmol^−1^h^−1^)	30.1 ± 0.9	30.7 ± 0.3
Μ (mCmol Cmol^−1^h^−1^)	100.9 ± 0.2	99.9 ± 0.6
q_CO2_ (mmol Cmol^−1^h^−1^)	74.6 ± 5.4	73.2 ± 1.4
q_O2_ (mmol Cmol^−1^h^−1^)	70.0 ± 5.4	70.4 ± 1.0
q_Acet_ (mmol Cmol^−1^h^−1^)	2.7 ± 0.1	5.7 ± 0.6
Residual glucose (mM)	0.183 ± 0.002	Max: 0.460 ± 0.01 Min: 0.094 ± 0.005
RQ	1.065	1.040

^a^ Reconciled specific rates at chemostat phase (D = 0.1 h^−1^) according to the approach described in Verheijen [18]; ^b^ Reconciled specific rates based on the approach of Verheijen [18] after integration and averaging over the cycle.

Nevertheless, the slight decrease in biomass yield found in this study was much smaller compared to the 25% decrease observed by Van Kleeff *et al.* [5] with a different strain and a residual glucose concentration of 0.15 mM. A decrease in the biomass yield (about 10%) was also observed for other microorganisms such as *E. coli* [19]. They reported that the cellular response of *E. coli* to repeated short-term glucose excess was a transient increase of glucose consumption and acetate formation. The formed acetate was not accumulated but consumed again when the glucose concentration reached low levels. Furthermore, they concluded that the formation of acetate, as an overflow reaction in zones with excess glucose concentration (about 7 mM in that study), is one important example of a rapid metabolic response to such zones, and suggested that it is reasonable that such a permanently repeated change of the environmental conditions may not only have short term regulatory effects on the metabolic level, but also long-time consequences for the culture. In our experiment with *S. cerevisiae* we observed a fast, but small production of acetate (Figure 1B, middle) during the first 20 s (feeding phase) and its concentration remained constant until about 180 s when it started to decrease back to the initial level (433 ± 50 uM). This finding may be an indication of acetate consumption when extracellular glucose concentration dropped (about 200 s). Finally, the averaged acetate production rate for the feast/famine culture (5.6 mmol Cmol^−1^ h^−1^) was higher compared to the reference steady state cultivation (2.7 ± 0.1 mmol Cmol^−1^ h^−1^).

### 2.4. The Feast/Famine Platform Has Interesting Features for Studying In Vivo Kinetics under Dynamic Conditions

For kinetic modeling it is important to obtain data from perturbations with a broad range of concentrations and fluxes. Besides, the motivation to mimic large-scale bioreactor conditions, the feast/famine setup is an advantageous perturbation system for *in vivo* kinetic studies. In contrast to stimulus response experiments (SRE) where a single perturbation is given, it is possible to monitor the dynamic metabolism during repetitive transients, from high to low substrate availability and vice versa. This feature, together with the high sampling frequency that can be obtained from repetitive samples, is perhaps the most powerful advantage of this setup with respect to other approaches commonly used to recreate dynamic conditions (e.g., step change in dilution rate, single pulse and periodic oscillations). Thus, in this study we were able to cover a wide range of substrate and metabolite concentrations and uptake/secretion fluxes in a time window of seconds by providing a fast, repetitive and reproducible perturbation in substrate concentration.

Among the different SREs reported in the literature, the pioneering work of Theobald *et al.* [6], where a single perturbation was given (in that case a glucose pulse), continues being the most appealing approach for studying metabolism under dynamic conditions. Single pulse perturbations are usually related to substrate availability (e.g., glucose, ethanol), to the transport of a particular molecule across the cell membrane (e.g., organic acids). Though highly informative when studying sudden perturbations to a particular system, the single pulse SRE approach may not recreate the actual conditions of a repetitive dynamic environment as is the case in natural and industrial environments. Thus, the metabolic response in a single SRE may be subject to an unforeseen scenario where the cells may not have the machinery ready to cope with such conditions. On the other hand, cells that are continuously exposed to perturbations, usually the case in reality, may have adjusted their metabolism in order to respond adequately to the dynamic environment they live in.

Theobald *et al.* [6] and Mashego *et al.* [9] applied single glucose pulse perturbations to a yeast culture growing at substrate limited steady state conditions to determine the metabolic response of the cells to a fast perturbation in a time window of few seconds (Table 2). While the secretion of by-products was apparently the primary observable response to the single glucose pulse, during the feast/famine experiment less by-products were secreted. In addition, Table 2 shows that the higher the glucose concentration achieved during the perturbation, the more by-products were secreted, most probably due to overflow mechanisms. For instance, the presence of ethanol is likely to be the result of a limited oxygen uptake capacity exhibited by *Saccharomyces cerevisiae*, which is known to be a Crabtree positive yeast [20]. In our study it is likely that the respiratory capacity was not exceeded with the applied gradients in substrate. Nevertheless, we have observed secretion of acetate even during the steady-state, which may point at some residual activity of pyruvate carboxylase and acetaldehyde dehydrogenase at both, steady-state and dynamic conditions.

**Table 2:** Comparison of four different stimulus-response experiments with aerobic cultures of *S. cerevisiae.*

Variable	Theobald *et al.*, 1997	Mashego *et al.*, 2006	Van Kleeff *et al.*, 1996	This study
Glucose perturbation	Pulse in STR	Pulse in STR/Bioscope	Block-wise feeding to STR	Block-wise feeding to STR
Strain	CBS7336	CEN.PK113-7D	CBS8066	CEN.PK113-7D
Dilution rate (h^−1^)	0.1 (before pulse)	0.05 (before pulse)	0.05 (average)	0.1 (average)
Min/Max. extracellular glucose concentration (mM)	0.07/5.5	0.11/2.8	~0/0.15	0.094/0.46
Observed by-products	Ethanol, Acetate, Glycerol	Ethanol, Acetate	None	Acetate
Min/Max qS (mmol/Cmol/h)	33/190	Not reported	Not reported	5/90

### 2.5. Dynamic Metabolic Response of S. cerevisiae to Feast/Famine Perturbations

Though it is one first observation since it takes place in the extracellular space, the secretion of by-products may not be the only difference between single pulse and feast/famine experiments. In the following, the metabolic profiles will be discussed comparing to former experiments of Theobald *et al.* [6] and Mashego, *et al.* [9] to highlight the main differences in the metabolic response between single pulse SREs and the feast/famine approach, and perhaps to show the main advantages of the latter. The observed dynamic metabolic response is presented in Figure 2, Figure 3, Figure 4, Figure 5, Figure 6, Figure 7 and Figure 8, where most of the intracellular metabolites showed a dynamic concentration profile following the extracellular glucose concentration pattern (Figure 2). However, as will be discussed later, some significant differences in response time and dynamics over the cycle were present indicating the action of some regulatory mechanisms.

#### 2.5.1. Extracellular Glucose Uptake Dynamics, the Entry Gate to Central Metabolism

The glucose uptake dynamics plays a vital role in the yeast’s growth on glucose [21]. As the entry gate to central metabolism, the glucose uptake is a variable that should always be considered. Thus, the extracellular glucose profile in the feast/famine showed a response similar to any other pulse experiment. However, the rate at which the glucose concentration changes in time followed a linear pattern. During the first 20 s (feeding phase), the extracellular glucose concentration increased from 0.094 ± 0.005 to 0.46 ± 0.01 mM. From 20 s onwards the glucose concentration dropped in time in a nonlinear fashion showing that the glucose uptake decreases with the glucose concentration. Surprisingly, the glucose was not fully depleted by the end of the cycle, which means that the cells did not experience an actual starvation phase. The residual glucose at the end of the cycle was 0.094 ± 0.05 mM, which was about half of the value in the reference chemostat at a dilution rate 0.1 h^−1^ (0.183 ± 0.002 mM).The maximum residual glucose concentration during the perturbation (0.460 mM in this experiment) was lower compared to the maximum obtained in the perturbation performed by either Theobald *et al.* [6] or Mashego *et al.* [9] with maximal glucose concentration of 5.5 and 2.8 mM respectively.

Unfortunately, the parameters of a Michaelis-Menten kinetic function for the glucose uptake in this experiment could not be identified because the residual glucose was always lower than Km, even for high affinity transporters, whose Km is about 1–10 mM [22,23]. The broad flat minimum space for the sum of squares only allowed for the estimation of a lower boundary of the Km = 0.9 mM, which is in agreement with literature values. Assuming that glucose transport follows a Michaelis-Menten-type mechanism and using the value of Km = 1 mM reported by Van Dijken *et al.* [24] for a culture running at similar conditions, we calculated a q_S,max_ value of 279 mmolCmol^−1^h^−1^. With these parameters, the dynamics of the glucose uptake can be estimated (Figure 2A). This result for q_S,max_ was in agreement with values reported in literature (237.6–316.8 mmolCmol^−1^h^−1^, [24]; 264.5 mmolCmol^−1^h^−1^, [25]). The Michaelis-Menten model of q_S_ *vs.* C_S_ deviated at low concentrations (Figure 3B). While the model predicts that the uptake would only stop when the concentration is equal to zero, the experimental measurements showed that concentration was never below 0.094 ± 0.05 mM. The reason for this behavior is still under investigation. Recently, Youk and Van Oudenaarden [21] argued that yeast’s growth on glucose cannot be explained solely based on glucose uptake, but a combination of extracellular glucose sensing and uptake. Whether or not the level of residual glucose in this experiment was playing a role in signaling cannot be answered here. To consider the minimum glucose concentration a linear relation (Figure 2B) for the uptake was used:

*q_S_* = *k*(*C_S_ − C_S,min_*), with k = 214.6 L Cmol^−1^ h^−1^ and C_S,min_ 0.05 mM (1)

It was found that at low glucose concentrations, the linear model provided a better representation of the kinetics, however, there was still a deviation from the observed measurements. Without using the feast/famine setup it might be impossible to observe this behavior. Indeed, other SREs focus on the rapid change in substrate availability and the following transient metabolic response, but these setups usually fail in providing sound information when restoring the initial status of the microorganisms. With the feast/famine it might be possible to recreate a before/after scenario due to the repeatability of the perturbations, and thus, it may provide additional information for a better understanding of the metabolism.

#### 2.5.2. The Energy and Redox Dynamics during the Cycle: With the Feast/Famine the Typical ATP Paradox Is not Observed

Typically, the increase in glucose concentration during single pulse experiments lead to a rapid increase in metabolic fluxes, and an initial decrease of the ATP concentration [6,9]. An interesting observation is that the sum of nucleotides (ATP + ADP + AMP) decreases after the pulse, which is referred to as the ATP paradox [26,27]. On the contrary, under the repeated pulse conditions applied here, where C_S_ changed from 0.094 to 0.46 mM, the ATP concentration increased directly after the addition of substrate to the reactor (Figure 3A). In addition, there was hardly a change in the sum of ATP, ADP and AMP (Figure 3F), whose values remained at a level comparable to the steady state (12.34 ± 2 μmol/gDW). Two hypotheses can explain this different response, 1- the achieved maximal glucose concentration was not high enough to trigger the ATP salvage mechanism; 2- the microorganisms became “trained” for the dynamic transients in such a way that they increased their oxygen uptake capacity to handle the dynamic environment without the need of an ATP salvage pathway usage. With the observed glucose uptake increase of about 9-fold in only 20 s, the microorganisms catabolized and immediately generated ATP reaching a new state with an increased ATP/ADP ratio (e.g., the energy charge increased from 0.8 to 0.9 in 40 s, Figure 3E), which would lead to an increased anabolism [28]. As it was expected for CEN.PK strains that contain a mutation in the adenylate cyclase [29], in this experiment we did not detect cAMP.

It is worth to mention that the maximal glucose concentration in this study was about 5 to 15-fold lower than in the single pulse experiments of Mashego *et al.* [9] and Theobald *et al.* [6], respectively. This suggests that the occurrence of the ATP-paradox might be dependent on the maximal glucose concentration reached. In addition, the high O_2_-uptake capacity realized in the repeated pulse cultivation clearly add to maintain a high ATP production capacity. The thermodynamic driving force (TDF, expressed as 1- Q/Keq) of the adenylate kinase reaction (Figure 3D) showed a fast increase of the TDF during the first 40 s indicating a fast regeneration of ATP from AMP, which in its turn is likely to be produced from an increased protein synthesis rate (*i.e.*, growth rate).

Upon an increased glucose flux, it is expected that the cell’s redox couple NAD/NADH becomes more reduced due to the dehydrogenation of glucose in the glycolysis and the TCA cycle. During the feast/famine the NADH and NAD whole cell concentrations showed a correlation mirroring the ATP profile (Figure 4) with NAD decreasing in the first 40 s. The NAD/NADH ratio was similar to the ratio of Aspartate/Malate, which has been reported to be a redox indicator in *P. chrysogenum* by Nasution *et al.* [30] and in respiring yeast [31] as an extension of the malate-oxaloacetate shuttle. The microorganisms seemed to be in a more reduced state during the reference steady state than under the feast/famine conditions as suggested by the lower NAD/NADH ratio. However, based on whole cell measurements Canelas *et al.* [32] reported a steady state value of NAD/NADH = 7.1 ± 2.7 (D = 0.1 h^−1^), which was similar to the one during the feast/famine. As expected, the total pool of redox cofactors remained nearly constant during the cycle (*i.e.*, NAD + NADH = 2.1 μmol/gDW and NADP + NADPH = 1.4 μmol/gDW). Note that the sum of NADH+NAD during the feast/famine remained close to the steady-state value, while the sum of NADPH + NADP was lower. Nevertheless, the NADP/NADPH ratio was comparable during the two conditions, *i.e.*, 0.21. With the feast/famine, the homeostatic properties of AXPs and NAD(P)H pools were observed even though the glucose uptake rate changed about 9-fold.

### 2.6. Possible Metabolic and Regulatory Mechanisms

#### 2.6.1. The Dynamics of Metabolites of the Glycolysis and the Pentose Phosphate Pathway

As expected, the concentration of metabolites in the upper glycolysis increased with an increasing glucose uptake rate (Figure 5), nevertheless, a delay of about 40 s in the concentration maxima was observed with average concentrations during the cycle comparable to the reference steady-state value. This apparent lack of correspondence between the extracellular glucose that peaked at 20 s, and the time at which the intracellular metabolites reached their maximum, suggests that some processes other than the glucose uptake or extracellular concentration may play a role. While in Mashego *et al.* [9] and Theobald *et al.* [6] it was observed that FBP increased right after the pulse and remained constant at high levels, in our observations we found that FBP also followed the pattern of the extracellular glucose. The phosphofructokinase reaction was reported to be activated by a decreasing ATP levels and increasing F6P, AMP and ADP concentrations [6]. However, in this study the metabolite response was different to that of the single pulse where a decrease in ATP was observed, while F6P increased. These observations suggest that the metabolic behavior of yeast is different when the cells are exposed to a repetitive perturbations, which is usually the case in reality, rather than that of a sudden pulse to a well-defined steady state cultivation (see also the previous discussion on ATP).

A remarkable dynamic behavior was observed with 3PG,2PG, PEP and to a certain extent with pyruvate. Whereas 3PG and 2PG showed a modest increase during the first 20 s of the cycle, PEP decreased for about 60 s, which corresponded to the concentration maximum observed for FBP. Although this behavior can be explained by an activation of the pyruvate kinase reaction [6], the metabolites of the lower glycolysis began to accumulate 60 s after the pulse, most likely following the falling pattern of FBP. Interestingly, from about 220 s onwards their concentrations decreased again, even though the FBP concentration was already at low levels. The observed behavior suggests that at metabolite level, there may still be unknown regulatory mechanisms controlling the dynamic response of the lower glycolysis and so, the carbon flux. Pyruvate concentration showed an almost constant concentration and about half of the steady-state value. This apparent absence of dynamics could be due to compartmentalization or a buffer capacity of the closely related amino acid alanine, which had a concentration about 100 times higher than pyruvate.

In the case of the pentose phosphate pathway (Figure 6), concentrations of the non-oxidative branch, with the exception of S7P and E4P, suggested a strong correlation with the upper glycolysis following the behavior of F6P. In the oxidative branch, although 6PG showed a behavior that followed its precursor G6P (open circles), there was a more notorious shift in the time for the maximum concentration to be reached (about 90 s) compared to G6P. In addition, the 6PG concentration remained high until 180 s, whereas G6P immediately dropped after its peak. Theobald *et al.* [6] observed a similar behavior in 6PG after a glucose pulse, interpreting this response as a delay that most likely followed a mechanism to keep FBP and GAP concentrations at high levels, e.g., the glycolytic flux increased faster than in the PPP.

In this study, we consider a metabolic delay when the substrate and product of a particular reaction respond concomitantly to the perturbation, but differ in the time the maximum is reached (*i.e.*, “peak-shifting”). This pattern can be clearly observed in 6PG reacting almost simultaneously with its precursor G6P, but reaching a maximum later, most likely due to a lower flux through this reaction. On the other hand, we observe a putative regulatory delay when there is a “lack of response” to changing substrate concentrations, such as the one exhibited by Ribu5P or X5P during the first 20 s where there was hardly a response. We speculate that this absence of immediate response indicates the presence of a putative regulation (most probably post translational modification), which could not be seen without a setup like the feast/famine.

#### 2.6.2. Dynamics of the Storage Carbohydrate Intermediates

It is frequently reported that the storage carbohydrates react rapidly to dynamic conditions, even under mild perturbations [33], thus during the feast/famine it might be expected these metabolites to exhibit a strong dynamic response. The metabolism of two major storage carbohydrates, trehalose and glycogen, was monitored through their intermediates (Figure 7). G6P is a precursor for G1P as well as T6P, with the former being involved in the synthesis and degradation of glycogen and the latter related to the synthesis of trehalose [34]. G1P followed its precursor dynamics, indicating that the phosphoglucomutase reaction was functioning at near-equilibrium as reported previously by Canelas *et al*. [35]. On the other hand, one can note that the dynamic response of T6P showed a delayed reaction, most likely due to a putative regulation. It is observed that the maximum concentration was reached at about 100 s, with a clear delay (*i.e.*, no response) of about 30 s in the beginning of the cycle. Though the mechanism of this putative regulation cannot be clarified from this data set, some indications have been previously suggested [36]. *In vitro* kinetic properties of T6P synthase were reported to show a weak affinity to G6P (3.5–5 mM). Since the levels of G6P reached the order of the affinity constant at about 20 s, this may explain the observed delay.

Although we obtained an excitation of about 10-fold in T6P, there was hardly a change in its product trehalose, which remained almost constant during the whole cycle. The nearly constant concentration of trehalose during the cycle seems to be in contradiction to what has been reported previously for glucose pulse experiments [7,33]. Although the concentrations did not change, there can still be a net flux that involves a simultaneous synthesis and degradation of trehalose. Trehalose was a large pool (when compared to other metabolites) with a concentration about 160 μmolgDW^−1^ at steady-state or 126 μmolgDW^−1^ during the feast/famine. The net decrease in trehalose concentration (about 30 μmolgDW^−1^) from chemostat to feast/famine would account for a decrease in the biomass concentration of about 1%. Though small, this change can partially explain the 5% lower biomass yield obtained during the feast/famine if we consider a possible similar change in glycogen concentration, which was not measured in this experiment.

Another example of a regulation that is not based on allosteric effects is observed with UDPG that showed a clear delayed dynamics and did not follow its precursor G1P. In particular, there was a delay during the first 20 s of the cycle and the maximum concentration was only reached after 160 s. This delay clearly points to the involvement of a post translational modification (PTM) mechanism. UDPG is reported to be a key metabolite not only in the synthesis of trehalose [34] but also in the metabolism of glycogen, cell-wall synthesis and protein N-glycosylation [37], therefore, the dynamics of UDPG will influence these processes.

#### 2.6.3. Dynamics of the TCA Cycle Intermediates

Remarkably, the dynamic concentrations of TCA cycle metabolites (Figure 8) largely deviated from those of the glycolysis and PPP. This behavior was most likely due to a compartmentalization phenomenon as well as to an interaction with large amino acid pools. Acetyl-CoA, like pyruvate, did not exhibit strong dynamic changes during the cycle. Nevertheless, the concentrations of products derived from Acetyl-CoA (*i.e.*, the metabolites of the oxidative branch of TCA) changed about 2 fold. TCA metabolite concentrations increased moderately in time until a maximum at about 200 s after which they decreased again.

### 2.7. Thermodynamic Analysis of Different Biochemical Reactions

During a dynamic experiment, flux determination is highly challenging since metabolite concentrations and uptake rates change rapidly. Here we use a set of metabolic reactions to highlight some advantages of the feast/famine setup for understanding the function of a metabolic network through the behavior of single reactions. In particular, using thermodynamic approaches can help to formulate constraints when estimating fluxes. Especially, some reactions of the central metabolism have been reported to operate close to equilibrium [35]. For near equilibrium reactions, the displacement from the thermodynamic equilibrium is quasi linear with the flux carried by that particular reaction. Canelas *et al.* [35] proposed an approximate kinetic format that is linear with the thermodynamic driving force (TDF):

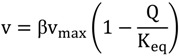
(2)
where v represents the metabolic (net) flux of the reaction, Q the mass action ratio, K_eq_ the thermodynamic equilibrium and representing enzyme specific properties (concentration, catalytic activity). Because the term cannot be measured directly, in this analysis we focus on the TDF change that is a proxy for the changes in metabolic fluxes. Note that reactions with additional allosteric or PTM regulation mechanisms will have a changing in time. For calculation of the ratios or conversion of intracellular amounts we have used the specific cell volume (1.7 mLgDW^−1^) and K_eq_ values reported in [35] or in [38].

**Figure 9:**
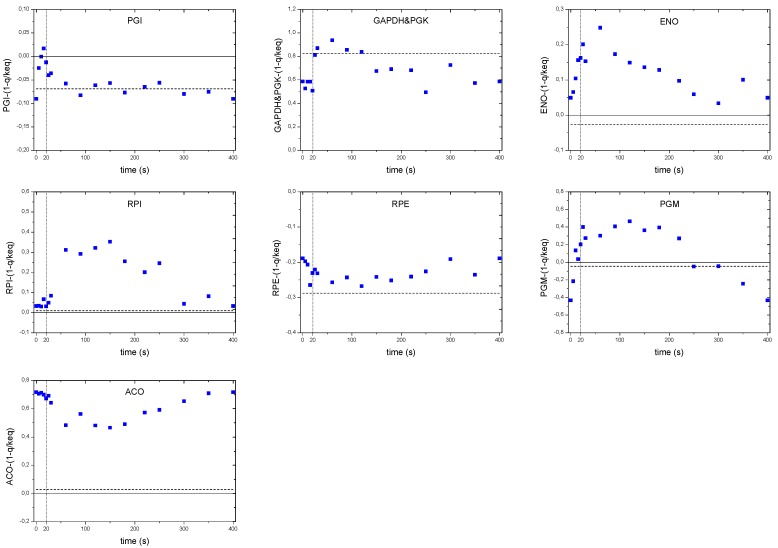
Thermodynamic driving force over the cycle for selected reactions. The dashed line represents the reference steady-state (D = 0.1 h^−1^). The vertical dotted line represents the end of the feeding phase.

As discussed in the previous sections, the concentration maxima of some intracellular metabolites were delayed compared to the extracellular substrate concentration. Nevertheless, the glucose uptake flux may be expected to peak (correlate) with the extracellular glucose concentration since the flux is usually a function of the concentration. Looking at concentrations only, there seems to be a disagreement between the intracellular concentrations and the metabolic flux of glycolysis that is directly linked to the glucose uptake reaction. This is reflected in the analysis of the thermodynamic driving force of the PGI reaction (Figure 9). While the substrate concentration peaked at 60 s, the TDF reached its maximum already at 20 s. After this maximum, we observed a steeper decrease in TDF than for the glucose uptake. This behavior might be due to other reactions consuming G6P, such as PPP, storage metabolism and biomass requirements. For the reactions downstream, like the ENO we did not observed a time delay in peak maxima.

Distinct flux behaviors were found in the case of the RPI and RPE reactions. While the thermodynamic driving force of the RPE reaction became more negative (*i.e.*, higher backward flux to the formation of Ribu5P), the TDF of RPI remained unchanged and close to zero for about 30 s, then a positive change was observed until about 300 s indicating a higher forward flux to the formation of R5P. Although, the reaction RPE seemed to run backwards during the complete cycle, the relative change in TDF was relatively small.

ACO, the second reaction of the oxidative branch of the TCA cycle, showed an unexpected pattern. By inspection of the TDF alone, the flux should decrease during the first 150 s and then increase until the end of the cycle. Being the only equilibrium reaction of the oxidative TCA, it is difficult to validate whether there was indeed a decreasing flux (*i.e.*, a behavior opposite to the glycolytic flux) or was an effect due to an extra phenomena like compartmentalization. A more interesting pattern was observed in the case of the storage pathway. In particular, the thermodynamic driving force of the PGM reaction suggest that during the first 20 s the PGM functioned towards the production of G6P (negative TDF). This behavior may originate from an active degradation of glycogen that would create a carbon flux to G1P, which enters the glycolysis via PGM. With an increased G6P concentration, the direction of the reaction would change towards the storage synthesis. At about 250 s, the TDF again turns negative indicating glycogen degradation until the end of the cycle.

## 3. Experimental Section

### 3.1. Strain and Culture Conditions

A cryopreserved stock culture (glycerol, −80 °C) of the haploid yeast *Saccharomyces cerevisiae* CEN PK 113-7D obtained from the *Centraalbureau van Schimmelcultures* (Fungal Biodiversity Center, Utrecht, The Netherlands) was used in this study. All cultivations were performed using a low-salt Verduyn minimal medium [39] with a glucose concentration of 7.5 gL^−1^. No ethanol was added to the medium because oscillations were not observed in these experiments. Microorganisms from one cryovial were used for the seed culture and grown for 10 h in 1L-Erlenmeyer flasks containing 100 mL medium at 200 rpm and 30 °C. The pre-culture was used to inoculate a 7 L bioreactor (Applikon, Schiedam, The Netherlands) containing a working volume of 3.894 L of broth. The aeration was performed using pressurized air at 0.992 L min^−1^ (approx. 0.25vvm) and a stirrer speed of 600 rpm. The broth was maintained at pH 5.0 by adding either 4M KOH or 2M H_2_SO_4_, the temperature was controlled at 30 °C and the pressure was kept at 0.3 bar (above atmospheric pressure). After the batch phase was complete (indicated by a strong and fast CO_2_ decrease and dissolved oxygen (DO) increase to almost saturation), the chemostat phase (steady-state) was started at a dilution rate of 0.1 h^−1^ for 53 h. After about 5.3 residence times, sampling for extra and intracellular metabolites at steady-state was performed.

### 3.2. Feast Famine Setup

**Figure 10:**
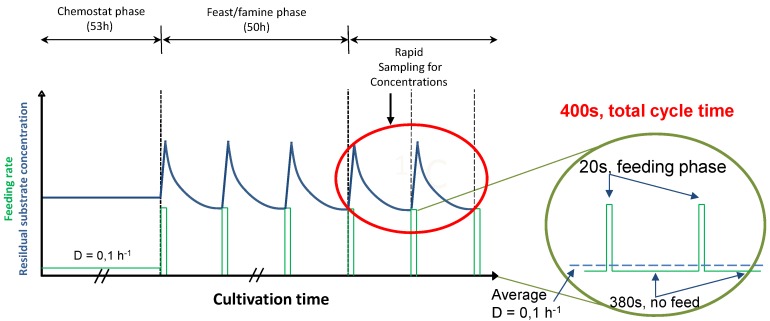
Profile of the experimental feeding and the resulting substrate concentration during the chemostat and the feast/famine phase. The feeding rate is represented by the solid green line.

After about five residence times of continuous feeding, the regime was changed to block-wise feeding in order to start the repetitive feast/famine phase (Figure 10). The feeding regime was designed to provide in average the same amount of substrate in time as in the previous steady-state period, but now added in a block-wise manner. The medium feed was switched on for 20 s at a rate 20 times higher than during the continuous phase using an automatic timer (PTC-1A, Programmable timing controller, Omega Engineering Inc., Stamford, CT, USA). In the following 380 s, no feed was added leading to a cycle length of 400 s. During the 20-second feeding period, 43 ± 1 mL of fresh medium were added. The same volume was then withdrawn during the first 260s at a flow rate of 0.166 ± 0.001 mL s^−1^ maintaining the broth volume nearly constant (max. 3.894 + 0.043 L).

### 3.3. Acquisition, Processing and Analysis of Samples

Broth samples for both extracellular and intracellular metabolite concentration measurements were rapidly withdrawn using two separate sample ports from the vessel during two subsequent cycles at corresponding cycle times (5 s, 10 s,…*etc.*) to obtain duplicate measurements.

#### 3.3.1. Extracellular Metabolites

For the measurement of extracellular metabolite concentrations, 1.5 mL of broth were withdrawn into a syringe containing pre-cooled (−20 °C) stainless steel beads (approx. 26 g). The cooled broth (~1 °C) was immediately filtered as described by Mashego *et al.* [9]. Extracellular (filtrate) glucose, ethanol, acetate and glycerol concentrations were determined by one/all of the following three methods: GC-MS, HPLC or enzymatic as described in [35]. We found that glucose determination by HPLC was not sufficiently precise for concentrations below 0.5 mM. Based on a series of samples with known concentrations, HPLC, enzymatic and GC-MS methods were compared showing that the enzymatic and the GC-MS methods performed well for concentrations below 0.5 mM with the latter being the most reliable method when the concentration was below 0.2 mM (data not shown). For GC-MS analysis, 20 μL of ^13^C cell extract was added to 100 μL of filtrate and transferred into a glass vial. The mixture was immediately frozen and kept at −80 °C until further processing, when the IDMS method described in Cipollina, *et al*. [40] and Wahl, *et al*. [41] was used for analysis.

The biomass concentration (dry weight) was determined by a gravimetrical method: 15 mL of broth were filtered through a pre-dried and pre-weighed membrane (Supor-450, 0.45 μm, 47 mm, Pall Corporation). The biomass-containing filters were then dried at 70 °C for 72 h and cooled to room temperature in a desiccator before weighing.

The O_2_ and CO_2_ volume fractions in the off-gas were measured by a combined paramagnetic/infrared NGA2000 analyzer (Rosemount Analytics, CA, USA). To account for transport and mixing effects due to the presence of head space and tubing, the approach described in Bloemen *et al.* [15] was used for estimating the time-dependent (at second-time scale) carbon evolution rate (CER) and oxygen uptake rate (OUR). An adaptation of the approach was applied in such a way that the kLa was maintained constant, while the DO was calculated when coupling liquid and gas-phase mass balances for O_2_ and CO_2_. An extended Kalman-filtering method was applied to estimate the time-dependent O_2_ and CO_2_ rates during the 400 s-cycle.

#### 3.3.2. Rate Reconciliation

The specific rates in chemostat and feast/famine cultures were reconciled following the approach described by Verheijen [18]. The reconciliation process is based on the application of the element conservation to find a better estimate of the rates. Initially the rates can be calculated from the measured concentrations of glucose in both the feed and the broth, the biomass concentration and the off-gas CO_2_ and O_2_ concentrations. The best estimate of the rates is then obtained by a least-square approach.

#### 3.3.3. Intracellular Metabolites

For the measurement of intracellular metabolite concentrations, 1.0 mL-broth samples were rapidly withdrawn and quenched in 5 mL cold (−40 °C) pure methanol [39] followed immediately by vigorous vortexing. The rapid sampling setup and procedure described by Lange, *et al*. [42] was used with some adaptations according to Douma, *et al*. [43] to achieve effective washing of the biomass. Thoroughly mixed cold broth/methanol samples were rapidly weighed, poured into a filtration device (with a Supor-200 cellulose membrane, 0.2 μm, 47 mm, Pall Corporation) containing 15 mL cold methanol (−40 °C). Subsequently vacuum was applied followed by an immediate washing step with 15 mL cold methanol (−40 °C). The filter containing the washed biomass was then transferred into a 50 mL-falcon tube (BD Biosciences) containing 30 mL of preheated (75 °C) aqueous ethanol solution (75% *v*/*v*). 100 μL of ^13^C cell extract were added to the tube as an internal standard [44]. The tube containing the sample was then tightly closed, shaken vigorously and placed into a water bath at 95 °C for 3 min for metabolite extraction. The tubes were then cooled using an ice bath and the filter removed. The remaining extract was concentrated later by complete evaporation of the ethanol-water mixture under vacuum as described by Mashego, *et al*. [45]. The dried residues were resuspended in 500 μL milliQ water and centrifuged at 15,000 g for 5 min at 1 °C. The supernatant was then transferred into a centrifugal filter unit (Ultrafree MC-ML, Millipore, MA, USA) and centrifuged at the same conditions. The filtrate was poured into a screw-capped plastic vials and stored at −80 °C until further analysis. Samples were analyzed by GC-MS [40,44,45] and/or LC-MSMS [46].

## 4. Conclusions

In this work, we propose a platform that uses a block-wise feeding regime for the identification of metabolic states (*in vivo* kinetics and regulation) under conditions similar to those encountered in nature as well as industrial large scale cultivations. This study shows that this regime (1) leads to reproducible metabolic responses; (2) allows to monitor the response to a disturbance from substrate limitation to excess and back to limitation; and (3) provides dense sampling (distributed through different cycles). The applied perturbation in extracellular glucose concentration (9-fold change) produced an excitation of intracellular metabolite concentrations up to 50-fold. We observed a slight decrease in the biomass yield (about 5%) as compared to the reference steady-state (D = 0.1 h^−1^) which can be partially explained by a higher acetate production rate (2-fold change) and a lower intracellular trehalose content (−20%). During these substrate perturbations, the typical ATP paradox response reported for single pulse experiments was not observed. An intracellular metabolic buffering and an increased oxygen uptake capacity of the “trained” microorganism could explain these observations but multi-omics techniques will be required to fully understand this behavior and confirm the hypothesis. In addition, the uncoupling of the substrate uptake rate and other extracellular rates (O_2_ and CO_2_) may be the result of dynamic intracellular metabolite accumulations.

Clear and distinguishable dynamic responses to a perturbation were observed for the different pathways analyzed (glycolysis, pentose phosphate, tricarboxylic acid and storage), amino acids (see Suplementary material), redox and energy cofactors. In particular, 6PG and T6P showed delayed responses compared to their precursor G6P suggesting the presence of regulatory mechanisms. In contrast, large amino acid pools (Ala, Glu, Asp) seem to buffer the dynamics of TCA metabolites and hardly react to the perturbation (almost constant concentrations). Under the conditions tested, some of the close to equilibrium reactions (e.g., GAPDH&PGK) showed changes in the product/substrate ratio up to 10-fold (about 5 kJmol^−1^ change in Gibbs free energy). These ratios were, in general, close to values reported previously. The thermodynamic driving force (TDF) of near equilibrium reactions was analyzed suggesting that flux changes are ahead of concentration changes, *i.e.*, maximal fluxes are not necessarily reached at maximal precursor concentration. Moreover, based on the TDF, we observed that the direction of some fluxes such as PGM changed under dynamic conditions. Thus, the feast/famine setup can also be used as a tool for identifying those flux changes which in turn can help in establishing constraints for flux estimations.

An experimental setup should aim at identifying *in vivo* kinetic properties, a challenge that has been approached through a number of modeling strategies [47,48,49,50]. High quality measurements, dense sampling and sufficient excitation are therefore essential for the success of this type of studies. The experiment presented here fulfills these requirements and can be used for a further extension with ^13^C labeling [11,47], and help to generate hypotheses on regulatory mechanisms like post translational modifications (PTM).

## Figures and Tables

**Figure A1:**
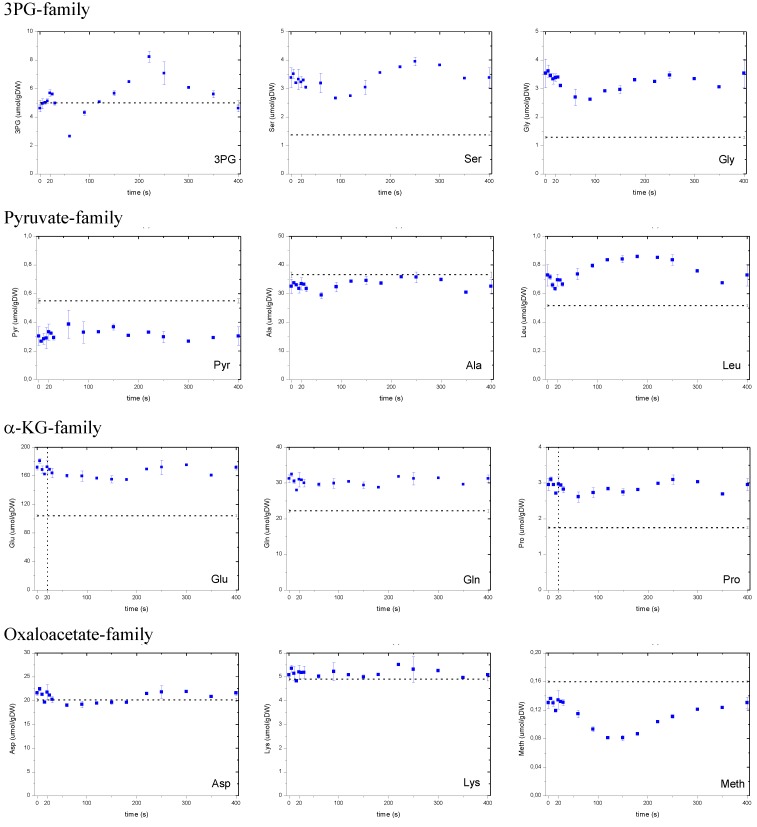
Concentration measurements of amino acids during the feast/famine cycle. The dashed line represents the reference steady-state (D=0.1 h^-1^), error bars represent the standard error of duplicate determination during two subsequent cycles. The vertical dotted line represents the end of the feeding phase.

## Abbreviations

### *Metabolites*

2PG: 2-phosphoglycerate
3PG: 3-phosphoglycerate
6PG: 6-phospho gluconate
α-KG: oxoglutarate
ALA: alanine
ASP: aspartate
CoA: coenzyme-A
DHAP: dihydroxy acetone phosphate
E4P: erythrose-4-phosphate
F6P: fructose-6-phosphate
FBP: fructose-1,6-bis-phosphate
FUM: fumarate
G1P: glucose-1-phosphate
G6P: glucose-6-phosphate
GAP: glyceraldehyde-3-phosphate
GLN: glutamine
GLU: glutamate
GLY: glycine
Iso-Cit: isocitrate
LEU: leucine
LYS: lysine
MAL: malate
METH: methionine
OAA: oxaloacetate
PEP: phospho-enol-pyruvate
PRO: proline
PYR: pyruvate
R5P: ribose-5-phosphate
Ribu5P: ribulose-5-phosphate
SER: serine
S7P: sedoheptulose-7-phosphate
SUC: succinate
T6P: trehalose-6-phosphate
UDPG: UDP-glucose
UDP: uridine-5-diphosphate
UTP: uridine-5-triphosphate
X5P: xylulose-5-phosphate.

### *Enzymes and*/*or the reactions they catalyze*

ACO: aconitate hydratase
AK: adenylate kinase
ENO: phosphopyruvate hydratase
FBA: fructose-bisphosphate aldolase
FMH: fumarate hydratase
G6PDH: glucose-6-phosphate dehydrogenase
GAPDH&PGK: glyceraldehyde-3-phosphate dehydrogenase + phosphoglycerate kinase
GPM: phosphoglycerate mutase
PFK: 6-phosphofructokinase
PGI: glucose-6-phosphate isomerase
PGM: phosphoglucomutase
PMI: mannose-6-phosphate isomerase
PYK: pyruvate kinase
RPE: ribulose-phosphate 3-epimerase
RPI: ribose-5-phosphate isomerase
TPP: trehalose-6- phosphatase
TPS: Trehalose-6-Phosphate synthase.

### *Other*

CER: Carbon evolution rate
DO: dissolved oxygen
IDMS: Isotope dilution mass spectrometry
OUR: Oxygen uptake rate
PPP: pentose phosphate pathway
SRE: Stimulus response experiment
TCA: tricarboxylic acid cycle
PTM: post translational modification
RQ: respiratory quotient.

## Appendix A. Supplementary Information

### A.1 Dynamics of Amino Acid Concentrations

The amino acid concentrations during the feast/famine are higher compared to the reference steady-state (with two exceptions, alanine and methionine). Their dynamics are coupled to their precursors, and much more damped because amino acids (Figure A1) are known to originate from metabolites (precursors) of the central carbon metabolism. This is the case of serine and glycine with their precursor 3PG (all about 4 umol g DW^-1^) which exhibit a similar dynamic pattern suggesting the presence of thermodynamic near equilibrium.

During the feast/famine, alanine does not follow the dynamics of its precursor pyruvate and the concentration of both precursor and product are lower compared to the reference steady-state. Although alanine may be present in different compartments (cytosol and mitochondria) it can still play a buffering role for the small pool pyruvate.

Glutamate, glutamine and proline are part of the α-KG family. They exhibit a similar time profile but differing from their precursor α-KG. The very high concentration of glutamate (180 μmol g DW^-1^) could play a role in buffering dynamics of the TCA cycle. Contrary to what has been observed for the pyruvate family all members of the α-KG family have a higher concentration during the cycle compared to steady state conditions. Nevertheless α-KG itself has a lower concentration. Amino acids belonging to the oxaloacetate family remain nearly constant over the cycle at concentrations close to the steady state values.
